# Radiobiological and Treatment-Related Aspects of Spatially Fractionated Radiotherapy

**DOI:** 10.3390/ijms23063366

**Published:** 2022-03-20

**Authors:** Leyla Moghaddasi, Paul Reid, Eva Bezak, Loredana G. Marcu

**Affiliations:** 1 Department of Medical Physics, Austin Health, Ballarat, VIC 3350, Australia; leyla.moghaddasi@austin.org.au; 2 School of Physical Sciences, University of Adelaide, Adelaide, SA 5001, Australia; eva.bezak@unisa.edu.au; 3 Radiation Health, Environment Protection Authority, Adelaide, SA 5000, Australia; paul.reid@sa.gov.au; 4 Cancer Research Institute, University of South Australia, Adelaide, SA 5001, Australia; 5 Faculty of Informatics and Science, University of Oradea, 1 Universitatii Str., 410087 Oradea, Romania

**Keywords:** non-conventional radiotherapy, GRID radiotherapy, protons, organ sparing, therapeutic index

## Abstract

The continuously evolving field of radiotherapy aims to devise and implement techniques that allow for greater tumour control and better sparing of critical organs. Investigations into the complexity of tumour radiobiology confirmed the high heterogeneity of tumours as being responsible for the often poor treatment outcome. Hypoxic subvolumes, a subpopulation of cancer stem cells, as well as the inherent or acquired radioresistance define tumour aggressiveness and metastatic potential, which remain a therapeutic challenge. Non-conventional irradiation techniques, such as spatially fractionated radiotherapy, have been developed to tackle some of these challenges and to offer a high therapeutic index when treating radioresistant tumours. The goal of this article was to highlight the current knowledge on the molecular and radiobiological mechanisms behind spatially fractionated radiotherapy and to present the up-to-date preclinical and clinical evidence towards the therapeutic potential of this technique involving both photon and proton beams.

## 1. Introduction to Non-Conventional Radiotherapy Delivery Approaches

**Figure 1:**
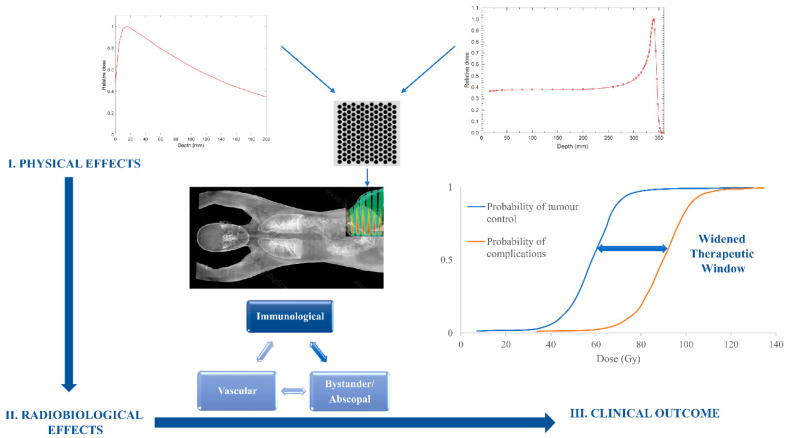
Schematic representation of the physical, radiobiological, and clinical effects concerning spatially fractionated radiotherapy delivered via photon or proton beams.

Radiation therapy is an effective cancer treatment and can be indicated as a form of therapy for up to 50% of cancer patients [1]. Most commonly, it is delivered using megavoltage photon and electron beams produced by medical linear accelerators. However, in recent times, there has been a rapid rise in proton beam facilities and some that use carbon beams to treat cancers. It is, though, not just the type of the radiation beams used that may impact the patient outcome in terms of tumour control and the development of side effects. These outcomes also depend on how the beam is delivered to a patient (i.e., broad, narrow, or even micro in size), the absorbed dose delivered (including dose per fraction, dose rate (hypo, hyper, or FLASH)), and even whether the tumour is irradiated uniformly simultaneously (volumetric irradiations), or using a scanning beam (a composition of 1D/2D dose paintings of the tumour), or non-uniformly irradiated, using spatial fractionation where only subvolumes of tissue and tumours are exposed during a treatment session. Irradiation of subvolumes only allows for larger radiation doses to be administered, which may otherwise be prohibitive in conventional volumetric radiotherapy due to the resultant toxicities of the surrounding healthy tissues, including the skin. As such, spatial fractionation may offer alternative approaches to treatments of larger and radioresistant tumours where higher radiation doses are necessary to achieve better treatment outcomes. The fact that radiation damage and tumour cell kill and/or side effects in normal tissues are dependent on the delivery techniques (including spatial and dose fractionation and dose rate) indicates that complex local radiobiological, as well as abscopal processes take place as a result of irradiation, including vascular damage and alterations, the response of the immune system, and even radiation-induced bystander effects in non-irradiated cells (Figure 1).

There has been a recent interest in spatially fractionated radiation therapy (SFRT), which can be implemented through different approaches, known as GRID [2], lattice [3], SBRT PATHY [4,5] (standing for PArtial Tumour irradiation targeting exclusively HYpoxic tumour segments), and even microbeam radiotherapy (MRT) [6] techniques. While there have been numerous articles describing these techniques, the current paper concentrated on the radiobiological mechanisms behind SFRT and summarizes the preclinical and clinical evidence of SFRT outcomes using photon and proton beams.

## 2. Molecular and Radiobiological Mechanisms of Spatially Fractionated Radiotherapy

Spatially fractionated radiation therapy was first used to exploit the advantage of skin-sparing effects in patients while enabling a high dose rate to refractory tumours in palliative treatments. Potential use of GRID, or other applications of SFRT, such as MRT, beyond the original palliative applications, and even multi-technique approaches [7,8] have been investigated due to findings of a greater therapeutic index resulting from both the increased normal tissue sparing and the greater tumour toxicity when compared to conventional temporally fractionated radiation therapy [9,10,11,12].

The use of the linear-quadratic (LQ) model to predict cellular responses to single high-dose fraction radiotherapy may, however, not be suitable given that it is based on clonogenic survival following much smaller and temporally accumulated doses. For radiosurgery, another single high-dose radiotherapy application, reports of tumour control outcomes are often underestimated using the LQ model [13]. Reduced normal tissue toxicities have been reported at high dose rates using SFRT in both animal and clinical studies. Findings from work by Dilmanian et al. [14] suggest the extent of normal tissue sparing to be greater than might be predicted simply by the volume effect that results from less normal tissue being directly irradiated. This suggests other biological responses may be at work where tissue is irregularly exposed in a series of beams that results in peaks and valleys of absorbed dose. The underlying biological mechanisms of response to SFRT are presently unclear, and most evidence on tumour and normal tissue response has come from animal studies. While more work remains to explain biological pathways and factors of influence, observed radiobiological effects from SFRT have been largely grouped into the interrelated areas of vascular, immunological, bystander, and abscopal effects [10].

### 2.1. Vascular Effects

Growth in tumour blood supply is driven by strong angiogenic signalling to meet the high metabolic demands of tumour tissue. The resulting vasculature differs, however, from normal tissue vessels, being aberrant in both structure and function [9,15]. Tumour endothelium is of an immature phenotype that supports quick regeneration, but also mediates therapeutic resistance by actively blocking immune responses to cancer cells following radiation therapy [15,16]. These developmental differences in tumour and normal vessels have come to light in animal studies of MRT that report preferentially toxic effects on tumour vessels. In the comparison of MRT effects on tumour and normal tissue by Bouchet et al. [17], histological analysis showed that phenotypically immature tumour vessels exhibited a loss of endothelium to the extent of denudation and destruction of the capillary plexus within microbeam paths. The mature vascular structure of normal tissues, however, displayed negligible or no detectable effects [17,18,19].

Biological responses to single high-dose irradiation (>8–10 Gy) such as with SFRT were described by Fuks and Kolesnick [20] as mediating endothelial radio-sensitisation that was not observed in temporally fractionated radiation therapy. With conventionally fractionated doses of 1.8–3 Gy, the resulting reactive oxygen species (ROS) were found to induce translation of preformed hypoxia-inducible factor (HIF-1) mRNA transcripts. Ensuing upregulation of pro-angiogenic elements was observed, including of vascular endothelial growth factor (VEGF), which interrupts normal apoptotic pathways and works to facilitate radioresistance [16,21].

Following single high-dose exposures, a different biological pathway was observed. Above a threshold of 8–10 Gy, responses in tumour endothelium were found to include translocation of the enzyme acid sphingomyelinase (ASMase). This enzyme is responsible for hydrolysis of sphingomyelin to phosphocholine and the proapoptotic messenger ceramide. Ceramide is a lipid found in cell membranes and plays a critical role in the radiation-induced apoptosis of endothelium [22]. In support of these findings, correlations were observed between sphingomyelinase activity and ceramide concentrations in patients receiving high-dose SFRT, as investigated by Sathishkumar et al. [23]. Serum samples from 11 patients receiving SFRT of 15 Gy were taken 24 h, 48 h, and 72 h following irradiation. Of patients showing partial or complete response to SFRT, six of eight demonstrated significant elevation in sphingomyelinase activity and ceramide levels and a positive correlation in clinical outcome (R^2^ = 0.818) [23].

### 2.2. Immunological Effects

Findings from several preclinical studies using various animal models reported that the highly heterogenous dose deposition achieved with SFRT is associated with a superior immunological response in tumour tissue [24,25]. Tumour cell ablation from areas of peak dose is thought to discharge tumour antigen material that enables dendritic priming of T-cells. Lymphatic cells are then able to enter tumour tissue via the conserved perfusion of the low-dose areas [3].

Greater immunological responses following MRT treatment have also been reported by Potez et al. [26] looking at melanoma mouse models. An MRT peak/valley dose of 408 Gy and 6.2 Gy was compared with a conventional broad beam irradiation of 6.2 Gy. Elevations of monocyte-attracting chemokines CCL2 and CCL5 were observed by enzyme-linked immunosorbent assay after MRT and were found to result in a substantial inflow of macrophages, natural killer cells, and importantly, CD4+ and CD8+ T-cells [26]. Furthermore, an immunohistological study of multidirectional MRT and broad beam irradiation of 9L gliosarcoma tumours in murine models by Eling et al. [27] showed that MRT supported a significant elevation of immune cell (CD68 macrophage) infiltration not seen with the conventional irradiation. This immune activity was found in both marginal and central areas of tumours, at 7 d and 14 d post irradiation [27].

Using reverse transcription quantitative polymerase chain reaction (RT-qPCR) and the RNA microarray, early responses in gene expression following MRT have been found to be largely modifications associated with inflammation and immunity [28]. Transcriptomic studies of 9L gliosarcoma cells in rat models examined modifications post MRT that showed adaptations associated with heightened inflammation and immune response. Following an MRT peak dose of 400 Gy and a valley dose of 18 Gy, the activation of pathways associated with NK cells and CD8+ T-cells was recorded [17,28]. Further studies that compared radiation-induced transcription changes between MRT and broad beam irradiations found gene expression following MRT to be differentially upregulated in favour of immunological responses. Overexpression of genes after MRT (including *Ccl9* and the human leukocyte antigen gene complex (HLA) family members responsible for encoding the major histocompatibility complex (MHC) Class II. *Ccl9* expression) is responsible for the CCL9 chemokine known to recruit immune cells to tumour sites and also found to have an immunological role in signalling antileukemic responses [25,29]. Importantly, these transcriptomic studies give evidence of different genetic pathways of response to radiation exposure, dependent on MRT or conventional broad beam irradiation, suggesting a possible biological mechanism behind the greater efficacy observed in tumour control with SFRT.

### 2.3. Bystander and Abscopal Effects

The effect on cells induced by radiation, in cells that are not directly traversed by radiation, but are adjacent to irradiated cells, is known as the bystander effect. Abscopal effects, on the other hand, refer to radiation-induced changes in cells distant from the exposed anatomy such as lymph nodes or metastatic sites. Bystander effects in terms of SFRT are considered to be the resulting changes in cells in low-dose areas between the beam tracks. Intracellular gap junctions, as well as extracellular soluble mediators are known functional pathways in this mechanism, but the exact nature of molecular signalling is not clear [30,31]. Evidence of abscopal effects is limited and largely anecdotal, but the effects are thought to result from differing pathways involving immunological agents [9,32,33,34].

A study by Peng et al. [35] comparing broad beam irradiations of both tumour and normal tissue, with different spatial arrangements of MRT, found the bystander response to be sensitive to the dose gradient. Survival fractions from across different irradiation fields were determined by a clonogenic assay. Cells exposed to a steeper dose gradient, where beams were spaced by 2.5 mm, showed a significantly smaller survival fraction of tumour cells when compared to conventional broad beam irradiation or MRT with beams spaced at 5 mm. Cell toxicity across the low-dose areas is thought to result from bystander signalling transmitted from the high-dose areas where steepness in gradient affects the transmission and distribution of signalling [35].

### 2.4. Proton SFRT

As compared to photon radiotherapy, the short physical range of protons, as well as the depth dose profile characterized by the Bragg peak offer a therapeutic advantage, especially in regard to greater normal tissue sparing, which results in the reduction of both early and late side effects. This is owed to the lower entrance dose of protons and the minimal exit dose, which renders them ideal for anatomically challenging locations. Furthermore, the highly localized dose distribution offered by proton beams allows dose elevation, which is greatly needed for radioresistant and aggressive tumours. Additionally, the radiobiological effectiveness (RBE) of protons is slightly higher than that of photons, and an RBE of 1.1 is currently being adopted for use in clinical practice.

Based on the above, the use of protons in SFRT applications presents another potential for even greater normal tissue sparing using the characteristic dose fall-off of particle radiation, distal to treatment volumes. This feature of proton therapy has been suggested to extend the use of SFRT to patients excluded from photon SFRT due to the proximity of critical organs at risk [36].

To this end, Mohiuddin et al. [37] reported clinical results of proton SFRT among 10 patients with different, but advanced and refractory cancers, unsuitable for photon SFRT. By the median follow up of 5.9 mo post proton SFRT, seven patients survived and three patients were deceased. Of the conditions among survivors, 5 patients had partial response, 1 had complete response, and 1 patient was stable. These results are reported to be equivalent to outcomes from photon SFRT and the type and extent of side effects similar in profile [37].

A direct comparison of photon and proton SFRT, with broad beam exposures, was performed in an in vitro study using a three-dimensional human skin model to test normal tissue damage. Normal tissue viability was determined by MTT testing. Comparing the results of the different radiobiological applications, both photon and proton SFRT were found to be equally sparing of the skin model, and both were significantly superior to broad beam application. That no radiobiological difference between proton and photon SFRT was evident in cellular responses suggests that the mode of irradiation, i.e., broad beam versus SFRT, may have a more substantive effect than radiation type [36].

## 3. Preclinical and Clinical Evidence with Spatially Fractionated Radiotherapy

### 3.1. Spatially Fractionated Radiotherapy with Photons

Aiming to improve the therapeutic index in photon radiotherapy, strategies have been devised utilizing radiobiological differences between normal tissue and tumour cells. High-dose spatially fractionated radiotherapy and ultra-high-dose-rate techniques have been explored. The two main variants of SFRT with photons that have been studied and implemented include GRID radiotherapy (GRID RT) and lattice radiotherapy (LRT)—a 3D configuration of GRID, delivering spherical subvolumes of a high dose within the tumour. Ultra-high-dose-rate methods, including FLASH and microbeam radiotherapy, and LRT are beyond the scope of this review and can be found elsewhere in the literature [2,10,31,38,39,40].

Spatially fractionated GRID RT with kilo- or ortho-voltage photons was originally developed by Alban Köhler in 1909 [41]. In the absence of skin dose modulation techniques, orthovoltage beams deposited the maximum dose at the surface, restricting dose escalation for the treatment of deeper-seated tumours. This technique was developed to allow dose escalation to palliate otherwise unreachable tumours while maintaining skin dose at a tolerable level. The reason for reduced skin injury is that discrete subvolumes of skin and subcutaneous tissues shielded under the grid serve as regrowth centres. With the advent of medical linear accelerators producing megavoltage (MV) photon beams and the emergence of modern treatment techniques with skin-sparing modulations without a grid, the original rationale for the use of SFRT with GRID was negated.

In the early 1990s, the use of MV photon beams modulated with GRID to deliver large mono- or hypo-fractionated doses of radiation regained momentum. Although skin toxicity was no longer an issue using MV photon beams and intensity-modulated techniques, megavoltage GRID relied on the superior repair potential of normal tissue in general, not exclusively skin, to allow taking advantage of high tumour control as a result of high-dose irradiation. The technique was generally used for bulky and deep-seated tumours before a conventional fractionation regimen or symptomatic palliative cases.

Understanding the mechanisms underlying observed high tumour response and low normal tissue complications using this technique has been the focus of ongoing research. As detailed in the previous section, the radiation-induced signalling effects and molecular pathways resulting from high-dose irradiation are different. The robust tumour response can be associated with the bystander effect [42,43,44], damage to endothelial cells lining blood vessels [23,45,46], and modulation of adaptive immune response (i.e., radiation is known to be immunosuppressive) [47]. For patients receiving GRID RT as a form of neoadjuvant treatment, tumour debulking resulting from high-dose irradiation encourages improved tumour reoxygenation [48], hence enhanced tumour radiosensitivity, for subsequent fractionated RT.

Over the last three decades, three main aspects of GRID with photons have been investigated: dosimetry, commissioning, and quality assurance [49,50,51]; treatment planning system and dose prescription [52,53,54]; efficacy and safety. Recently the Radiosurgery Society (RSS) GRID/Lattice, microbeam and FLASH working group has published recommendations and guidelines for GRID RT with photons [54]. The report is the clinical physics consensus and guidelines to standardise this treatment modality in terms of technology selection, commissioning, QA, TPS approaches, response modelling, and so forth.

GRID was originally generated by commercially available cerrobend blocks, designed to be mounted into the gantry head. The blocks turned the broad beam photon beams into pencil beams to deliver a hexagonal pattern of hot and cold spots on the patient’s skin. Key parameters for the characterisation of non-uniform dose distribution by GRID are the peak and valley dose, referring to the dose along the unshielded and shielded beam paths, respectively. The ratio of these parameters, the valley-to-peak dose ratio (VPDR), quantifies the spatial fractionation and varies with the GRID model, beam energy, field size, and depth. The next-generation of GRID used modern multileaf collimators (MLC) and treatment planning systems (TPSs) to design virtual grid blocks [55,56,57]. This development has offered two main advantages: more flexibility in creating grid patterns, unlike block grids, which can only have one pattern; eliminating the need to mount/dismount heavy grid blocks. However, MLC-based GRID requires more monitor units (MUs), hence longer beam-on time, and a larger radiation leakage and surface dose. A hybrid collimation system was proposed [58] utilising both MLC and GRID blocks, each generating parallel stripes in orthogonal directions. The system requires fewer monitor units compared to MLC-based GRID and allows more flexibility compared to block GRID systems.

In the following subsections, within the context of photon GRID, we review the radiobiological formalism for tumour and normal tissue response evaluation, clinical data in terms of response rate and toxicity profiles, and mathematical-model-based response evaluation.

#### 3.1.1. Response Modelling in GRID RT with Photons

In clinical radiotherapy, the treatment planning evaluation has historically been performed by dose–response relationship parameters, tumour control probability (TCP) and normal tissue complication probability (NTCP). TCP is the probability of zero surviving clonogenic cells, and it is a function of the survival fraction (SF), which can be calculated using the linear-quadratic model. NTCP models are generally more complicated and depend on the absorbed dose and tissue-specific parameters.

In the era of 3D simulation and treatment planning, ICRU 83 [59] recommendations, which are based on a dose–volume approach for plan evaluation (treatment efficacy), are used. Over the recent years, an increasing number of clinical trials (RTOG (https://www.rtog.org/Clinical-Trials, accessed on 22 January 2022), ESTRO (e.g., [60]), etc.) have tried to correlate various treatment regimens to their corresponding toxicities and subsequently proposed plan objectives that balance treatment efficacy against normal tissue toxicity. In the meantime, the sturdiness of analytical radiobiological-based evaluation metrics has become subject to criticism due to their limitations and simplifications. Therefore, the use of such analytical formalisms has gradually been replaced in favour of evidence-based objectives. However, in an ever-evolving field of radiotherapy, analytical radiobiological evaluations remain very useful to design improved treatment protocols, predict the therapeutic index for techniques at the preclinical stage of development, and streamline patient selection in light of the expected biologic consequences (e.g., GRID).

The GRID dose distribution is highly inhomogeneous; therefore, for dose–response calculations, a single nominal/prescription dose value cannot be used for the entire population of tumour cells. The equivalent uniform dose (EUD), originally formulated in the 1990s for spatially nonuniform dose distributions [61], can be used. Apart from the dose, the use of the standard LQ model for high doses (>10 Gy for GRID) has been debated [62,63,64,65]. Brenner asserted the applicability of the LQ model for a high dose range [62]. Others argued that the LQ model overestimates cell kill efficacy for a high dose range as the radiosensitivity parameters have been obtained for the low-dose region and the effect of lethal misrepair is reduced for high dose delivery [63,65]. Current guidelines for GRID RT with photons [54] recommend using the modified LQ (MLQ) model, introduced by Guerrero [64]. The MLQ model expands on the LQ model by incorporating the repair rate and treatment and produces a better fit to iso-effect curves from several experimental datasets compared to the LQ model [64]. Zhang investigated the effect of the use of the LQ versus MLQ model for EUD and therapeutic ratio (TR) determination [66]. The effect was found to be minimal at 20 Gy per fraction dose with the calculated EUD and TR using the LQ model being 2.8% and 1% higher than those obtained by the MLQ model, respectively.

**Figure 2:**
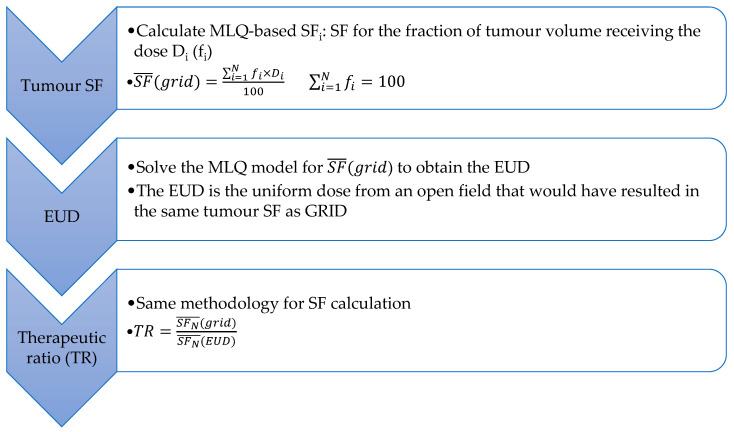
The diagram demonstrates the therapeutic ratio calculation process for GRID RT with photons [54].

The formalism [54], as demonstrated in Figure 2, postulates that the grid and open fields with the same EUD result in the same tumour cell kill efficacy. Therefore, the therapeutic ratio (TR) is defined as the normal tissue survival fraction ratio with GRID to that with an open field. A patient can benefit from the GRID technique if TR is greater than 1, that is lower normal tissue toxicity is expected with the same tumour control if SFRT is used.

#### 3.1.2. Mathematical-Model-Based Studies

The efficacy of GRID RT with photons has been investigated using mathematical modelling of treatment response.

Zwicker [67,68] used the LQ model to evaluate survival fractions of tumour and normal tissue in GRID RT with MV photons and a hexagonal cerrobend grid collimator. To incorporate the effect of penumbral dose in MV photon beams, the dose distribution at a certain depth was approximated by the superposition of radial dose functions centred on each local peak. Typical values of α/β ratios of 10 and 2.5 were assumed for tumour and normal tissue, respectively. The therapeutic ratio was determined using a methodology similar to that described in Figure 1, except with the LQ model. Cell sensitivities were modelled by SF_2_ (survival fraction at 2 Gy), and TRs were quantified as a function of tumour and normal tissue SF_2_, each ranging from 0.3 to 0.7 for three nominal doses of 10–20 Gy. The calculated TRs were from 0.88 to 13.22. It was concluded that SFRT GRID with photons offers a therapeutic gain in a wide range of cell sensitivities unless radiosensitive tumour cells reside in a more radioresistant normal tissue (TR = 0.88). The results suggested that the technique is most advantageous for radioresistant bulky tumours (e.g., hypoxic tumours) surrounded by radiosensitive normal tissue.

A similar observation was obtained in a more elaborate study by Zhang et al. [53]. The 2D dose distribution was calculated by an MCNPX Monte Carlo (MC) code, which was validated against EDR2 films and water tank measurements. TRs were calculated using the MLQ model for a range of fractionated GRID RT schemes, with individual α and β values corresponding to several melanoma cell lines and three normal tissue cell lines with varying degrees of radiosensitivities being used. The incorporation of cell-line-specific MLQ model parameters allowed for examining the sensitivity of TR to various responding cell lines. It was found that acutely responding tumours (α/β > 6) surrounded by radiosensitive normal tissue benefit the most from GRID RT. The TR values in this study ranged from 0.939 to 2.902. Follow-up work from the same group [69] reported on TR values for the conventionally fractionated (2 Gy/fractions) and hypofractionated (15 Gy/fraction) grid therapy for several cervical cancer cell lines residing within three normal tissue cell lines with different radiosensitivities. In the data analysis, the effect of fractionation and grid therapy was isolated. It was concluded that while there was a therapeutic gain for both conventional and hypofractionated schemes as compared to open field single fraction regimens, the true gain was expected for hypofractionated regimens. In addition, tumours surrounded by radioresistant normal tissue do not benefit from GRID RT (TR < 1). The effect of dose size in a single-fraction GRID RT for melanoma was the subject of the next study of this group [66]. The findings of this study suggested that to obtain the full benefit of this technique, a minimum of 15 Gy per fraction should be used followed by conventionally fractionated open field RT.

#### 3.1.3. Clinical Studies

A summary of clinical studies reporting on the response rate and normal tissue complications of GRID RT using photon beams is presented in Table 1. The first clinical findings on the efficacy and safety of GRID RT with megavoltage photon beams were reported by Mohiuddin’s group [70,71,72] for palliative patients with bulky tumours who had been treated to tolerance or were refractory to conventional radiotherapy. The first cohort consisted of 22 patients with diverse histologies in a palliative setting [70]. Treatment was delivered using a hexagonal grid block and MV photons delivering GRID doses ranging from 10 Gy to 15 Gy. The toxicities were analysed as acute and late complications and the palliative response rate (pain, mass, bleeding, or dyspnoea) as the complete (CR) or partial (PR) relief of symptoms. There was an impressive overall response rate of 91% (20/22 patients) with toxicities compared to conventional therapy, i.e., 14% mild acute and 9% late toxicities, with one patient requiring intervention surgery. The next study expanded to 61 patients in the same category as the previous cohort [72]. There were 6 MV and 25 MV beams used to deliver GRID doses of 10–20 Gy in a single fraction. A 91% overall response rate was observed (27% CR and 64% PR) with no acute morbidity. The results in this study were stratified for four different palliative symptoms. The follow-up report included the outcome of GRID RT for 63 patients for palliation of tumours with diverse histologies with or without subsequent fractionated RT and eight patients with head and neck (H&N) cancer for definitive treatment with GRID RT neoadjuvant to conventionally fractionated RT [71]. The overall response rate maintained a high rate of 91% for palliative cases with one reported Grade 3 mucositis and one fatal acute morbidity. Of eight patients who received GRID as part of definitive treatment, a clinical CR was observed in 62.5% of patients and a pathological CR was confirmed in 50% of patients. In the context of definitive radiotherapy, clinical CR is defined as the complete elimination of the tumour examined clinically or radiographically and clinical PR refers to the cases where a 50% reduction in the tumour size is observed in clinical/imaging examinations, usually 1 mo after the completion of the treatment. The pathological evaluation was conducted for four out of eight patients who underwent surgery following their courses of treatment, that is all the evaluated specimens were clear of residual disease.

The clinical evaluation of 6–20 MV GRID RT with or without subsequent conventional RT for a cohort of 19 palliative patients with melanoma on various sites was conducted by Kudrimoti et al. [73]. The overall response rate (local control) was 80% (seven CR, nine PR), and no severe toxicity (grade > 3) was observed. The overall survival improved for patients receiving subsequent fractionated RT of greater than 40 Gy as compared to less than 40 Gy. This observation supports the findings from mathematical evaluations from the study of Zhang et al. [66].

Sathishkumar investigated the response rate of GRID RT for 34 patients with diverse histologies and treatment sites from a molecular perspective [46]. The study aimed to find the possible correlation of cytokine enzymes (e.g., TNF-α), believed to be released as a result of high-dose radiation, in patients’ blood circulation with their tumour response rate. A strong correlation between clinical CR and TNF-α induction was demonstrated. The clinical CR and PR to GRID RT in this study was seen in 32% and 49% of the patients. The next work of this group studied the possible role of GRID RT (15 Gy/one fraction) in elevated levels of S-SMase serum and ceramide in 11 patients with various histologies and investigated the correlation of these levels to the treatment response rates [23]. It was concluded that GRID RT leads to increased levels of S-SMase serum and ceramide, which correlates with the clinical response. The overall response rate (CR + PR) was 74% (8/11), where 62% (5/8) of those presented with a substantially elevated level of serum (2–20-fold) post GRID RT. Studies of this type not only provide clinical evidence, but also present clinicians with prognostic factors that can be used as a guideline in patient selection procedures.

Huhn et al. [74] reported on a series of 27 patients with H&N cancers N2-3 treated with GRID plus fractionated RT (Group 1 = 14) or GRID plus fractionated RT followed by surgery (Group 2 = 13). Treatment evaluations were in terms of local neck control, pathological response (Group 2 only), overall and disease-free survival, and toxicities. Overall neck control was comparable between Groups 1 and 2, 93% and 92%, respectively. Disease-free and overall survival for Group 1 was 50% and 86%, respectively, at 44 mo follow-up, and 85% and 92%, respectively, at 116 mo follow-up for Group 2. Pathological analysis showed a CR of 85% and 15% PR. The local tumour control of 91% at three years was observed in a cohort of 53 patients with H&N cancer T4 and N3, treated with a single fraction 15 Gy GRID followed with conventional RT in a curative setting [81]. Normal tissue toxicities remained mild for most of the patients with 4% developing more than Grade 3 complications. These observations were reflected in a more recent publication that presented the results of their retrospective analysis on the efficacy and safety of GRID RT for 21 patients with bulky and advanced H&N cancer [82]. The treatment protocol consisted of single-fraction 15–20 Gy GRID followed by fractionated IMRT with or without systemic therapy in both palliative (12/21) and definitive (9/21) settings. The response rates for patients who completed more than 75% of their IMRT course (15/22) was 70% and 87.5% for the palliative and curative groups, respectively, with one Grade 3 and four Grade 4 acute skin toxicities.

Ten palliative patients with locally advanced NSCLC and treated by single-fraction 15 Gy GRID with a 10 MV beam followed by 60 Gy fractionated RT were followed up (2–24 mo, median 4 mo) for response evaluation and toxicities [75]. Complete alleviation of symptoms was observed in 71.4% of patients with 28.5% showing a partial response without any significant acute or late complications or morbidity.

Encouraging treatment outcomes have been reported for soft tissue sarcoma. Clinical investigations of treatment outcomes for 33 patients with advanced soft tissue sarcoma treated by a single fraction of 12–20 Gy of 6 MV GRID with or without subsequent conventional RT revealed an overall response rate of 76% with mild acute and late toxicities and three cases of Grade 3 skin reactions [76]. An interesting finding of this study, which supports predictions of the mathematical study of Zhang [66], is the significant improvement of tumour response for patients receiving subsequent RT of greater than 50 Gy (95% (45% CR, 50% PR)) as compared to those receiving lower than 50 Gy 59% (10% CR, 50% PR). The next report included 14 sarcoma patients treated with a consistent protocol of a single-fraction 18 Gy GRID followed by 50 Gy conventional RT concomitant with chemotherapy and surgery thereafter [80]. An impressive pathologic complete response rate (pCR) (>90% necrosis) of 65% was observed. Two patients did not complete treatment, one due to Grade 3 acute skin toxicity and the other limb amputation. Toxicities among the rest were mild with two with delayed wound healing. A dramatic tumour response of 90% was observed following 18 Gy single-fraction GRID followed by 32 Gy conventional RT for a case of high-grade spindle cell sarcoma, which was otherwise uncontrollable despite being on conventional RT with limb amputation as a prospect [79]. The incorporation of GRID RT before standard fractionated radiotherapy for patients with bulky soft tissue sarcomas has become a standard protocol in recent years. Snider et al. reported on the pCR (>80% necrosis) and safety of their institutional protocol consisting of single-fraction 15 Gy GRID followed by conventionally fractionated radiotherapy (45–50.4 Gy) for patients with bulky soft tissue sarcomas and various histologies [83]. A pCR rate of 35.3% with 50% in extremity disease was achieved. Although the pCR in this study was lower than that in Mohiuddin’s series, it exceeded the pCR rates of 19.4% and 27.5% reported for neoadjuvant chemoradiotherapy (RTOG 9514) or radiotherapy (RTOG 0630) for patients with localized soft tissue sarcoma, respectively [86]. A case study was reported of a patient with high-grade bulky (22 cm in the greatest dimensions) soft tissue sarcoma treated by 15 Gy GRID RT followed by 50 Gy fractionated RT [85]. The complete response was achieved with more than 50% radiological response and no greater than Grade 3 skin toxicities.

Clinical studies reporting on cohorts treated later than 2005 (the advent of the widespread use of digital linacs and multileaf collimators (MLC)) reflect a change in the technology of the GRID delivery from cerrobend grid blocks to MLC-generated grid fields. Fourteen patients with locally advanced and large H&N cancer treated with MLC-based GRID therapy to 20 Gy from a 6 MV beam in one fraction followed by 30 fractions IMRT and concomitant chemotherapy were evaluated for clinical or pathological response rates and normal tissue toxicities in a median follow-up of 19.5 mo [77]. An overall response rate of 79% was observed with acute skin and mucosal toxicities lower than Grades 3 and 2, respectively. One incidence of death due to carotid blow-in was reported. Skin toxicities in this study were more severe than those reported by Huhn et al. [74]. This can be attributed to the increased skin dose due to the higher MU required for an MLC-generated grid field. In a retrospective analysis of follow-up data from 79 patients with various bulky cancer types treated with GRID, the response rates from two techniques, cerrobend (39/79) and MLC-based (40/79) grid, were compared [78]. The response rates for palliative patients (61/79) showed no statistically significant difference between MLC-based and cerrobend-based GRID RT. The majority of toxicities were mild (Grades 1–2) with six (15%) Grade 3–4 acute skin toxicity in the MLC group as compared to one (5.1%) in the cerrobend group. However, the authors stated that the difference was not statistically significant. Although this study concluded there was no difference between response rate and toxicities between the two techniques, it should be noted that the cohort was quite diverse with a wide range of prognosis parameters. Pokhrel conducted a retrospective analysis of 13 patients with bulky and deep-seated tumours at various sites, treated with single-field cerrobend GRID RT in their department to test the efficacy and safety of the MLC-based technique [87]. The patients were replanned, and plan evaluation was performed using standard DVH-based metrics for the tumour, skin, and organs at risk. Their simulation demonstrated that the 3D conformal MLC-based GRID resulted in enhanced target coverage with reduced skin and organs at risk toxicity. Using a similar approach in a cohort of patients consistently with breast cancer, Murphy et al. demonstrated that the dosimetric qualities of MLC-based GRID plans were comparable with block-based GRID with the added advantage of superior conformity for MLC-based plans [88]. Plan evaluations were conducted in terms of the EUD and TR using DVH curves.

With the accumulation of clinical evidence indicating the efficacy and safety of GRID RT and other high-dose and high-dose-rate modalities, along with the ever-evolving technologies in the field of megavoltage photon radiotherapy allowing high-precision beam deliveries, the concept of the GRID has been expanded for the management of otherwise refractory malignancies. For example, recently, a volumetric modulated arc therapy (VMAT) planning approach for grid therapy was proposed and tested on two palliative cases as a proof of principle at the Mayo Clinic [84]. The approach included delivery of a high dose and dose rate (flattening filter-free (FFF) mode) to spheres within the GTV. VMAT provides more real estate allowing for dose delivery to complicated structures while maintaining the dose to OAR under the tolerance. This combined with superior tumour control and normal tissue preserving offered by GRID could further improve TR for patients with bulky and advanced disease.

Increasing evidence on the potential of megavoltage photon GRID radiotherapy for the management of advanced and bulky tumours, resulting in a heightened appeal of SFRT among clinicians, warrants the standardisation of these techniques. Randomised clinical trials, stratified by cancer sites and molecular subtypes, should be designed to assess and formulate curative or palliative endpoints. As discoveries move from the realm of conceptualisation to preclinical and clinical applications, and with improved clinical trial designs in the light of findings from radiobiological and molecular research, the future outcomes for patients with bulky advanced malignancies are deemed to improve.

### 3.2. Spatially Fractionated Radiotherapy with Protons

Based on the interest and preclinical success of spatially fractionated photon radiotherapy, the idea of merging proton therapy with spatial dose fractionation (or GRID therapy) has surfaced under the term “proton minibeam radiotherapy” and put into practice over the last few years by several research teams [54,89,90,91,92].

Proton minibeams are pencil beams or planar proton beams having sub-millimetre dimensions, which are administered in a spatial configuration separated by 2–4 mm gaps, resulting in a lateral dose profile of successive regions of high dose and low dose, appearing as peaks (delivering lethal dose) and valleys (delivering sub-lethal dose). A parameter of interest is the ratio between the two doses (peak-to-valley dose ratio), which is relevant for tissue sparing. The success of spatial fractionation relies on the dose-volume effect, whereby the redistribution of the dose into smaller than conventional fields enhances normal tissue tolerance. Tumour control is presumed to be similar to conventional proton irradiation, owing to the narrow scattering of protons with increasing depth, which leads to uniform irradiation of the target volume [93]. GRID therapy delivers an elevated tumoricidal dose in the high-dose regions, while also affecting cancer cells in the low-dose regions via non-targeted effects, such as bystander response.

#### 3.2.1. Animal Studies

While preclinical studies on proton minibeams are limited both as anatomical locations and numberwise, they show promising results and potential translation into clinics (Table 2). Studies were designed with various beam arrangements and array configurations, with different centre-to-centre beam distances to assess the influence of these parameters on the therapeutic index.

To evaluate acute normal tissue effects with proton minibeam versus broad beam irradiation, tumour-free BALB/c mouse ears were exposed to either 16 (4 × 4) minibeams with a nominal dose of 6 kGy or to homogenous broad beam of 20 MeV protons of 60 Gy [94]. Normal tissue effects were up to four-times more pronounced in the homogeneously exposed mice than in the minibeam group, showing significant ear swelling, erythema, and desquamation, but also hair loss and irreversible damage to the sebaceous glands, the latter effects lacking in the minibeam-irradiated group. The same group investigated the beam size dependence of normal tissue effects in proton minibeam irradiation when different beam widths were used [95]. They showed that mice ear skin reactions decrease with decreasing beam size not only superficially, but also in the deeper layers of the tissue. The dependence between beam width and tissue effects was so prominent that acute effects were completely evaded with 180 μm squared minibeams in a grid of a 1.8 mm centre-to-centre beam distance. Differences in normal tissue reactions between conventional and minibeam irradiation were suggested to be caused by the complex immune response and also vascular effects that are responsible for better normal tissue sparing with spatially fractionated proton beams. The study established that future technical advances should focus on minibeam sizes below 0.1 mm for ideal normal tissue response in both superficial therapies and deep-seated tumours.

An important aspect of proton minibeam therapy to widen the therapeutic ratio is treatment accuracy through both daily and total dose modulation in order to reproduce spatial reirradiation with high precision. In their recent report, Sammer et al. showed that an array shift between daily fractions that decreases the total peak-to-valley dose ratio can influence normal tissue sparing [99]. Their experiment on healthy mouse ear indicated that irradiation in the same position with four fractions of 20 MeV protons using an array of 16 minibeams with a centre-to-centre distance of 1.8 mm, a positioning accuracy of 110 ± 52 μm, and a peak dose of 314 Gy (high daily dose) resulted in the least severe toxicities, whereas a shift of the array by 0.9 mm (avoiding any previously irradiated tissue) led to considerable acute toxicities, an increase in fibrous tissue, and a higher otitis score. The most significant adverse effects, both acute and late, were observed with the employment of four fractions of the 64 proton minibeam array with a centre-to-centre distance of 0.9 mm applied to the same tissue spot with a peak dose of 78.6 Gy (low daily dose). Therefore, besides the beam positioning accuracy, another key factor that influences the outcome is the total dose modulation of microbeam arrays, showing that high dose modulation maintaining a low valley dose is more advantageous than low dose modulation with a low peak-to-valley dose ratio. If transposed into clinical settings, the aspect of accurate reirradiation of previously exposed regions becomes even more critical, as patient positioning and movement both intra- and inter-fraction is a constant concern in radiotherapy.

In their study series on glioma-bearing rats, Prezado et al. showed that proton minibeam therapy widens the therapeutic index for high-grade gliomas not only by reducing toxicity, but also by increasing tumour control [96,97,100]. Proton minibeam therapy significantly reduced neurotoxicity in rat brains as compared to standard proton therapy, as shown by a 6 mo follow-up [96]. Skin damage, radionecrosis, and severe astrocyte activation were observed in the standard group, while in the minibeam group, no skin damage was reported and only one rat presented with light lesions on microglial nodules (see the beam details in Table 2). In the study investigating tumour control in rats irradiated with minibeams versus untreated rats, Prezado et al. demonstrated a 22% tumour-free long-term survival, without notable normal tissue effects [97]. This observation suggests the possibility of dose elevation and/or changes in fractionation regimen to further increase tumour control in these aggressive brain malignancies. The results are even more remarkable considering that these results were obtained after a single fraction delivery of a highly heterogeneous dose. The impact of temporal fractionation was also evaluated in a subsequent study, where the initial radiation dose was administered in two fractions having the same biologically equivalent dose as the single fraction [101]. This resulted in 83% tumour-free long-term survival, which is 2.2-times higher than the survivals in the single-dose group and 3.3-times higher than the percentage of long-term survival after conventional proton irradiation, showing that temporal fractionation can further widen the therapeutic index of proton minibeam therapy in aggressive tumours.

As a follow-up of the previous experiments on the effect of proton minibeams on high-grade gliomas in rats, the preservation of key cerebral functions, such as motor, emotional, and cognitive functions was evaluated over 10 mo after whole-brain irradiation [98]. The results showed very good preservation of brain functions after proton minibeam irradiation, with only a slight reduction in body weight as compared to the unirradiated group, which might be indicative of some effects on the pituitary gland and/or hypothalamus. This aspect might require further optimisation of proton minibeam irradiation, in particular if clinical exposure is intended for paediatric patients where weight loss should be avoided.

The mechanisms responsible for the outcomes of proton minibeam irradiation are possibly related to cell signalling through non-targeted effects, strongly involving the immune system via CD8 T-cells. In normal tissue sparing, an important role is played by the fast repair of vascular damage.

#### 3.2.2. Clinical Studies

To date, the number of clinical reports on spatially fractionated proton therapy is few. The first report on proton GRID treatment was published in 2018 by Gao et al. after a feasibility study consisting of phantom-based simulations of both shallow- and deep-seated targets [102]. The two patients presented in the study were treated for retroperitoneal dedifferentiated liposarcoma and nasopharyngeal osteosarcoma, respectively. The employed GRID design was different from the proton minibeam settings of a submillimeter width often reported in animal studies. Here, the pencil beam scanning proton beam consisted of multiple energy layers, with the highest energy spots deposited at the most distal end of the target, while the lowest energy spots deposited at the most proximal end. The layer setting resulted in high-dose (focused spot) and low-dose regions (between spots). The anatomical location of the tumours and vicinity to critical structures rendered these patients ideal candidates for proton GRID therapy. Both patients received 15 Gy_(RBE)_ in a single dose, with minimal skin toxicity (Grade 1 radiation dermatitis). Calculated doses received by the organs at risk after proton GRID showed reduced normal tissue effects as compared to photon GRID therapy and a very good sparing of critical organs located beyond the target [102].

The first proton minibeam treatment plans were reported by Lansonneur et al. on two brain tumours—a high-grade glioma (three fields) and a meningioma (two fields) using patients’ CT datasets with delineated structures [103]. The aim of this preliminary study was to evaluate the spatial fractionation of the dose in normal tissues during treatment of deep-seated tumours and to ensure the maintenance of a homogeneous dose in very large targets (glioma PTV = 66.8 cm^3^). The two minibeam settings used were: (1) a 4 mm centre-to-centre distance to provide a uniform PTV dose distribution and (2) a 6 mm centre-to-centre distance to increase spatial fractionation in normal tissue (despite decreasing PTV homogeneity). Target dose homogeneity was evaluated via the sigma index, i.e., the standard deviation of the normalized differential DVH curve. The peak-to-valley dose ratios at a depth of 20 mm ranged from 9.2 to 12.8, larger than in previous animal measurements reported by the same group, showing further enhancement of the experimental setup with better normal tissue sparing and the feasibility of proton minibeams for clinical implementation. When compared to standard proton therapy, similar DVHs were obtained for deep-seated organs (brainstem), while proton minibeam treatment resulted in a significant decrease of the average doses received by shallow organs at risk. These preliminary results showed the advantage of spatial fractionation to deliver tumoricidal dose to the target while widening the therapeutic window through better normal tissue sparing.

#### 3.2.3. Heavy-Ion Minibeam Therapy

Due to their dose distribution characteristics similar to protons, but higher radiobiological effectiveness, heavy ions are considered to offer greater clinical benefits to cancer patients than proton therapy. Carbon, oxygen, and helium ions were therefore investigated in the form of spatially fractionated radiotherapy to evaluate their possible advantages over proton minibeam therapy [104,105]. The premise of these studies was that the enhanced dose deposition of ions could offer dose elevation for aggressive and radioresistant tumours, which together with the minibeam setting also reduces normal tissue effects.

Dosimetric characteristics of carbon and oxygen minibeams were evaluated using a prototype multi-slit collimator that was 700 μm wide line apertures with a 3500 μm centre-to-centre distance [104]. Carbon minibeams presented somewhat lower peak-to-valley dose ratios than oxygen minibeams, though for both beams, these values were significantly higher than in photon minibeams, showing a potentially better sparing of the normal tissue. The reduced lateral scattering for heavy ions led to a larger output factor in the centre of the spread-out Bragg peak than the one measured for proton minibeams. A disadvantage of carbon/oxygen ions is their beam fragmentation tail towards the distal end of the tumour, which is considerably less in helium ions, making them possible candidates for minibeam therapy.

A comparative dosimetric study between proton and helium minibeams was undertaken by Schneider et al. using Monte Carlo simulation in a water phantom and CT images of the human head [105]. The range of both minibeams was 7.7 cm, and different beam sizes with various beam spacings were used to evaluate the depth dose curves, peak-to-valley ratios, and linear energy transfer values. The study showed that for the same minibeam spacing, helium ions produced higher peak-to-valley dose ratios and greater Bragg peak-to-entrance dose ratios than protons. On the other hand, dose homogeneity in the target was harder to achieve with helium ions due to lower lateral scattering. While smaller mini beam spacing would address the homogeneity issue, this would compromise the peak-to-valley dose ratio in normal tissues. Pre-clinical studies are therefore required to evaluate the impact of target dose heterogeneity with helium minibeams on tumour control.

## 4. Conclusions

Radiotherapy has shown some great advancements over the decades owing to translational and interdisciplinary research across connected science fields. Radiotherapy delivery underwent an important evolution from conventional to non-conventional administration in order to tackle aggressive tumours and/or to offer higher protection to the normal tissue. Nevertheless, therapies with substantial impact on tumour control or normal tissue sparing are still in the preclinical phases or awaiting clinical validation.

High-dose spatially fractionated radiation has been clinically evaluated over the last few decades using various grid designs to allow for non-uniform dose delivery. With a similar concept, a more recently developed non-conventional irradiation technique employing minibeams via an array of closely spaced beams was appraised. This form of beam setting is thought to influence cell signalling via abscopal effects, leading to the difference in response between malignant and healthy cells. Delivery of spatially fractionated radiotherapy was trialled with photons and proton beams alike. While showing real potential to widen the therapeutic window, these techniques require a better understanding of the radiobiological and physico-chemical properties for further optimisation and broader clinical implementation. Further developments in non-conventional radiotherapy are therefore dependent on the multidisciplinary research undertaken at several levels: in silico, in vitro, and in vivo.

The latest developments in radiotherapy have shown the potential of “omics” fields to advance research and to shed more light on several molecular aspects behind cellular response to radiation. Given that a number of underlying mechanisms elicited by spatially fractionated radiotherapy are yet to be explained, omics research combined with the existing trends in GRID therapy could answer some of the persisting questions regarding the therapeutic advantage of this technique over conventional irradiation, in particular for aggressive tumours.

**Take home summary:**

- Spatially fractionated radiotherapy aims to widen the therapeutic window by better sparing of normal structures;
- While not all mechanisms behind this technique are fully elucidated, some potential biological mechanisms include: differential vascular effects between cancer and normal tissues, bystander or abscopal effects, and immunological effects;
- To date, both low-LET and high-LET radiation-based therapies have been investigated in vitro, as well as in vivo;
- Clinical studies are limited, nevertheless with promising results; further multidisciplinary research is required to clarify the radiobiological and physico-chemical mechanisms to understand and explore the full potential of spatially fractionated radiotherapy, in particular for aggressive and recurrent cancers.

## Figures and Tables

**Table 1:** Summary of clinical studies reporting on the response rate and normal tissue complications of GRID RT with photons. FRT: fractionated radiotherapy; CR: complete response rate; PR: partial response rate (50%); NI: not indicated; H&N: head and neck cancer; pCR: pathologic complete response.

Author (Year)	Number of Patients	Histology	Dose (Gy)	RT Regimen	RT Goal	Overall Response Rate% (CR%,PR%)	Complications
Mohiuddin (1990) [70]	22	Diverse	10–15	GRID	Palliative	91 (26,67)	5 mild acute, 1 mild late toxicities
Mohiuddin (1996) [72]	61	Diverse	10–20	GRID	Palliative	91 (27,64)	No severe acute toxicity/morbidity
Mohiuddin (1999) [71]	63	Diverse	10–20 GRID; 50–70 FRT	GRID and GRID + FRT	Palliative	91 (16,75)	1 Grade 3 mucositis, 1 acute morbidity
	8	H&N		GRID + FRT	Curative	100 (63,37)	No Grade 3 or higher
Kudrimoti (2002) [73]	19	Melanoma	12–20 GRID; NI	GRID and GRID + FRT	Palliative	80 (37,47)	No Grade 3 or higher
Sathishkumar (2002) [46]	34	Diverse	12–20 GRID; NI	GRID + FRT	Curative	81 (32,49)	-
Sathishkumar (2005) [23]	11	Diverse	15 GRID; 60 FRT	GRID + FRT	Curative	74	-
Huhn (2006) [74]	14	H&N	15–20 GRID; 54–79 FRT	GRID + FRT	Curative	Neck control: 93%	Acute and late toxicities of Grades 1–2
	13			GRID + FRT + Surgery		Neck control: 92% 100 (85,15)	
Somaiah (2008) [75]	10	NSCLC	15 GRID; 60 FRT	GRID + FRT	Palliative	(71.4,28.5)	No Grade 3 or higher
Mohiuddin (2009) [76]	33	Sarcoma	12–20 GRID; 22–70 FRT	GRID and GRID + FRT	Palliative	76 (26,50)	Mild acute and late toxicities; 2 Grade 3 acute skin reaction
Penagaricano (2010) [77]	14	H&N	20 GRID; 54–66 IMRT	GRID + Chemo + IMRT	Curative	79	Acute and late toxicities of Grades 1–3, 1 death carotid blow-in
Neuner (2012) [78]	39 cerrobend)	Diverse	10–20 GRID; 12–70 FRT	GRID and GRID + FRT	Palliative+ Curative	Pain: 75 (25,50) Mass: 67 (17,50)	Acute and late toxicities of Grades 1–2; 2 acute Grades 3–4
	40 (MLC)					Pain: 74 (30,44) Mass: 73 (9,64)	Acute and late toxicities of Grade 1–2; 6 acute and 3 late Grades 3–4
Kaiser (2013) [79]	1	Sarcoma	18 GRID; 32 FRT	GRID + FRT	Curative	90% tumour regression	No skin toxicity
Mohiuddin (2014) [80]	14	Sarcoma	18 GRID; 50 FRT	GRID + FRT + Surgery	Curative	pCR (>90% necrosis): 65%	1 Grade 3 acute skin reaction; 2 late wound healing
Edwards (2015) [81]	53	H&N (T4 & N3)	15 GRID; 48–79.2 FRT	GRID + FRT	Curative	91% clinical local control	4% > Grade 3 toxicity; 2 requiring feeding tube
Choi (2019) [82]	7	H&N	15–20 GRID; Variable	GRID + FRT	Palliative	70	1 Grade 3, 4 Grade 4 acute toxicity
	8				Curative	87.5 (44.4,13)	
Snider (2020) [83]	26	Sarcoma	15 GRID; 45–50.4 FRT	GRID + FRT	Curative	pCR (>80% necrosis): 35.3%	27% > Grade 3 acute skin toxicity
Grams (2020) [84]	2	Pancreas; Abdominal leiomyosarcoma	20 GRID; 20–30 FRT	GRID + FRT	Palliative	marked reduction in tumour size; symptomatic relief	NI
Tajiki (2021) [85]	1	Sarcoma	15 GRID; 50 FRT	GRID + FRT			

Abbreviations: NSCLC = non-small cell lung cancer; IMRT = intensity modulated radiation therapy; MLC = multileaf collimator.

**Table 2:** Compilation of animal studies investigating normal tissue effects and/or tumour control after proton minibeam irradiation.

Study Aim [Ref]	Treatment Protocol	Observations
Normal tissue toxicity evaluation after mouse ear irradiation		
Comparative study of mouse ear irradiation [94]	(1) Homogenous 20 MeV proton field of overall 60 Gy (2) Minibeam (4 × 4 minibeams, 0.18 × 0.18 mm^2^, 1.8 mm distance) of 6000 Gy peak dose	Up to 4-times greater extent of ear swelling, erythema, and desquamation in the homogenous group vs. minibeam. Hair loss and damage to the sebaceous glands observed only in the homogenously exposed group.
Minibeam size dependence of normal tissue effects in mouse ear [95]	20 MeV minibeam (4 × 4 minibeams, 1.8 mm centre-to-centre beam distance) of a 6 kGy peak dose, with various beam sizes	The ideal minibeam size for minimal side effects should be < 0.1 mm. Still, any spatial fractionation with a beam size < 1 mm leads to superior normal tissue protection as compared to homogenous irradiation.
Normal tissue toxicity and tumour control evaluation after high-grade glioma treatment in rats		
The effect of proton minibeam vs. standard proton therapy on rat high-grade gliomas [96]	(1) Standard proton therapy of 100 MeV with 2 Gy/min at a 1 cm depth of 25 Gy in one fraction (2) Proton minibeam of 400 μm-wide slits and 3200 μm centre-to-centre distance, 57 Gy peak dose at a 1 cm depth	Main aim: to evaluate the reduction in neurotoxicity with proton minibeam. Rats under standard proton therapy developed significant skin damage and long-term brain damage (after 6 mo of evaluation) as compared to minimal toxicity in the minibeam group.
Tumour control of high-grade gliomas in rats with proton minibeam [97]	100 MeV proton minibeam of 400 μm-wide slits and a 3200 μm centre-to-centre distance, 70 Gy peak dose at a 1 cm depth; one dose fraction	22% disease-free long-term survival (6 mo follow-up) in the treated group. Mean survival time of the control group (untreated): 18 d; mean survival time of minibeam group: 32.5 d.
Evaluation of cerebral functions in rats with high-grade gliomas after proton minibeam [98]	100 MeV proton minibeam with a 57 Gy peak dose at a 1 cm depth. Minibeam width at 1 cm was 1100 ± 50 μm.	No locomotory, behavioural, or cognitive differences observed between proton minibeam and control (unirradiated) groups. Small growth perturbation was observed in the treated group (12.5% lower body weight and size).
